# Isolation and characterization of a salt-tolerant denitrifying bacterium *Alishewanella* sp. F2 from seawall muddy water

**DOI:** 10.1038/s41598-020-66989-5

**Published:** 2020-06-19

**Authors:** Rui Cheng, Xinyi Wang, Hui Zhu, Baixing Yan, Brian Shutes, Yingying Xu, Baorong Fu, Huiyang Wen

**Affiliations:** 1 Key Laboratory of Wetland Ecology and Environment, Northeast Institute of Geography and Agroecology, Chinese Academy of Sciences, Changchun, 130102 P R China; 2 University of Chinese Academy of Sciences, Beijing, 100049 P R China; 3 Jilin Provincial Engineering Center of CWs Design in Cold Region & Beautiful Country Construction, Changchun, 130102 P R China; 4 School of Environment, Liaoning University, Shenyang, 110036 P R China; 5 Urban Pollution Research Centre, Middlesex University, Hendon London, NW4 4BT UK; 6 Key Laboratory of Songliao Aquatic Environment, Ministry of Education, Jilin Jianzhu University, Changchun, 130118 P R China

**Keywords:** Water microbiology, Environmental biotechnology

## Abstract

A salt-tolerant denitrifying bacterium strain F2 was isolated from seawall muddy water in Dalian City, Liaoning Province, China. Strain F2 was identified by morphological observations, physiological and biochemical characteristics and 16 S rDNA identification. The salt tolerance of strain F2 was verified and the factors affecting the removal ability of strain F2 to nitrous nitrogen (NO_2_^–^N) and nitrate nitrogen (NO_3_^–^N) in saline conditions were investigated. Strain F2 was identified as *Alishewanella* sp., named *Alishewanella* sp. F2. Strain F2 can tolerate NaCl concentrations up to 70 g/L, and its most efficient denitrification capacity was observed at NaCl concentrations of 0−30 g/L. In the medium with NaCl concentrations of 0−30 g/L, strain F2 exhibited high removal efficiencies of NO_2_^–^N and NO_3_^–^N, with the removal percentages for both NO_2_^–^N and NO_3_^–^N of approximately 99%. In saline conditions with 30 g/L NaCl, the optimum culture pH, NaNO_2_ initial concentrations and inoculation sizes of strain F2 were 8−10, 0.4−0.8 g/L and 5−7%, respectively. Strain F2 was highly effective in removing NO_2_^–^N and NO_3_^–^N in saline conditions, and it has a good application potential in saline wastewater treatment.

## Introduction

In the past 40 years, with the rapid development of aquaculture industry in coastal areas of China, a large amount of coastal aquaculture wastewater has been discharged, which brought about various negative impacts on the environment^1^. The coastal aquaculture wastewater usually contains both inorganic salts (on average of 10−30 g/L NaCl) and many contaminants including nitrogen^2^. Large amounts of nitrogen pollutants (i.e., nitrous nitrogen (NO_2_^–^N) and nitrate nitrogen (NO_3_^–^N)) which are continuously released from the uneaten feed residue can result in the increase of inorganic nitrogen pollution in aquaculture water year by year^1,3^. Furthermore, with the rapid development of industrialization, a large number of saline wastewater sources, e.g., vegetable pickled wastewater^4^, textile wastewater^5^, and oily wastewater^6^, etc., with NaCl concentrations exceeding 30 g/L have been produced in various industrial processes. Most saline industrial wastewater also contains a large amount of nitrogen pollutants^7,8^. The increasing discharge of saline wastewater from aquaculture and industry without any treatment has threatened the aquatic, terrestrial and wetland ecosystems^9,10^. Therefore, the removal of nitrogen pollutants from saline wastewater has become an urgent problem.

Nitrite, an intermediate product of nitrification and denitrification, is frequently detected in water bodies and seriously threatens the aquatic organisms and human health^11,12^. The concentration of nitrite in wastewater is up to 50 mg/L or more^11^. High concentrations of nitrite seriously endanger the growth and normal metabolism of aquatic organisms. Humans accidentally drinking water containing high concentration of nitrite could easily lead to impaired intelligence and form strong carcinogens in the human body (e.g., nitrosamines), and even lead to death. As the most stable form of nitrogenous compounds in the aerobic environment, nitrate has a high solubility and can migrate and diffuse rapidly in water, resulting in secondary pollution^13,14^. The discharging of untreated and unqualified wastewater containing nitrate into surface water bodies will bring serious potential safety hazards and threaten the growth of plants, animals and human health^15^. Therefore, effectively reducing the NO_2_^–^N and NO_3_^–^N concentrations in water is of great practical significance for both ecosystem and human health.

The main technologies for removing nitrogen from wastewater include physical, chemical and biological methods^16–19^. Compared with physical and chemical methods, biological methods have the advantages of high efficiency, low energy consumption, low cost, easy implementation, etc., and have been widely used to remove NO_2_^–^N from wastewater. Denitrification can convert NO_2_^–^N and NO_3_^–^N into gaseous nitrogen (i.e., nitrogen (N_2_) and nitrous oxide (N_2_O)), and fundamentally solve the nitrogen pollution problem in water^20^. Several nitrite-type denitrifying bacteria, e.g., *Acinetobacter baumannii*^21^, *Pseudomonas putida*^22^, and *Pseudomonas tolaasii*^23^, etc., have been screened and were proved to be efficient in removing NO_2_^–^N from wastewater. However, saline wastewater contains not only high concentrations of nitrogen but also a large amount of soluble salts. The presence of salts can significantly and negatively affect the removal of pollutants by microorganisms^24,25^. When microorganisms are exposed to highly saline environment, osmotic pressure is increased, resulting in the excessive loss of water in general microbial cells, the separation of protoplasm, the inhibition of microbial growth and metabolism, and even death^26^. The denitrification capacity of most denitrifying bacteria is inhibited by high salinity^27^. Therefore, salt-tolerant denitrifying bacteria with efficient NO_2_^–^N and NO_3_^–^N removal ability are required to be isolated for the treatment of saline wastewater.

The objectives of this study are to: 1) isolate and identify a salt-tolerant denitrifying bacterium strain F2 from seawall muddy water; 2) validate the salt tolerance as well as the denitrification capacity of strain F2; and 3) evaluate the effects of initial pH, NaNO_2_ initial concentration and inoculation size on the denitrification capacity of strain F2 in saline conditions. The results of this study will provide efficient microbial resource and optimal process parameters for microbial denitrification of saline wastewater, which is of great significance for protecting the water environment safety and human health.

## Results

### Isolation of denitrifying bacteria

An anoxic condition was created by submerged culturing of denitrification medium with NaNO_2_ as the sole nitrogen source, but a small amount of NaNO_2_ was still oxidized and rapidly converted into NaNO_3_^28^. After repeating three times of enrichment and isolation, three strains of denitrifying bacteria with nitrite as sole nitrogen source were obtained, and numbered F1, F2 and F3, respectively. The three strains became turbid during the enrichment and cultivation in the denitrification medium that was accompanied by different degrees of gas generation (i.e., N_2_, N_2_O and/or nitric oxide (NO))^29^. The enrichment cultivation of each strain cultivated for 5 d is shown in Fig. S1 (in supplementary material).

Table 1 shows the removal percentages of NO_2_^–^N and NO_3_^–^N by each respective strain. After a 5 d cultivation, the removal percentages of NO_2_^–^N in denitrification medium by all the three strains were above 98%. However, the removal percentages of NO_3_^–^N by strain F2 were higher than that of other strains. The removal percentages of NO_2_^–^N and NO_3_^–^N by strain F2 were 98.38% and 96.13%, respectively, and gas was produced concomitantly in a 5 d cultivation. During the 5 d of cultivation, ammonia nitrogen (NH_4_^+^-N) concentration in the medium of strain F2 increased slightly, and the total nitrogen (TN) removal percentage by strain F2 was 20.99% (Fig. S2 in supplementary material), which indicated that some of the NO_2_^–^N and NO_3_^–^N in the medium were transformed into NH_4_^+^-N, and some were transformed into nitrogen-containing substances which are necessary for the growth of strain F2 through assimilation. Besides, according to the conservation of elements, the removal of TN in the medium was mainly due to the emission of gaseous nitrogen (i.e., N_2_, N_2_O and/or NO). After comprehensive analysis, strain F2, an ideal bacterium, was selected as the research object in the subsequent experiments.

**Table 1:** Removal effect of NO_2_^–^N and NO_3_^–^N by strains in denitrification medium after 5 d cultivation.

Strain	NO_2_^–^N initial concentration(mg/L)	NO_2_^–^N effluent concentration(mg/L)	NO_2_^–^N removal percentage(%)	NO_3_^–^N initial concentration(mg/L)	NO_3_^–^N effluent concentration(mg/L)	NO_3_^–^N removal percentage(%)
F1	115.90 ± 4.63	1.43 ± 0.12	98.76	42.47 ± 1.19	6.43 ± 1.98	84.85
F2	106.87 ± 4.58	1.73 ± 0.54	98.38	45.60 ± 1.77	1.77 ± 0.54	96.13
F3	105.50 ± 3.09	1.37 ± 0.45	98.70	45.00 ± 2.30	4.33 ± 1.89	90.37

### Identification of strain F2

**Figure 1:**
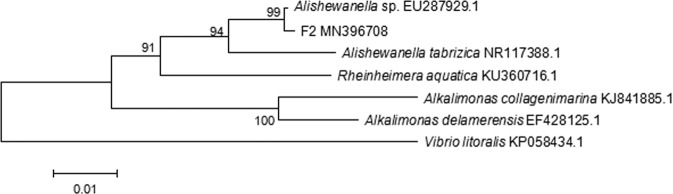
Molecular phylogenetic tree of strain F2 based on 16 S rDNA sequences.

The physiological and biochemical characteristics of strain F2 are shown in Table 2. The extracted bacterial DNA was amplified using 16 S rDNA primers to a 1033 bp amplified fragment (Fig. S3 in supplementary material). The phylogenetic tree constructed by the MEGA 4.0 version software is shown in Fig. 1. The 16 S rDNA identification reveals that strain F2 was 99.81% homology genetic related with *Alishewanella* sp. N5 (GenBank accession no. EU287929.1). Therefore, according to the morphological observation and 16 S rDNA gene analysis, strain F2 was identified as *Alishewanella* sp., named *Alishewanella* sp. F2 (GenBank accession no. MN396708). It was deposited at the China General Microbiological Culture Collection Center (CGMCC) on March 25, 2019, numbered CGMCC No: 17433.

**Table 2:** Physiological and biochemical characteristics of strain F2.

Characteristic	Result	Characteristic	Result
Gram’s stain	—	Metabolism	Facultative anaerobic
Glucose oxidative fermentation	Fermentation	Halotolerance (% NaCl)	0–7
Oxidase	+	Catalase	+
Nitrate reduction	+	Denitrification	+
Indol test	—	H_2_S test	+
M.R test	—	V.P. test	—
Gelatin hydrolysis	+	β-galactosidase test	—

Note: “+“ means positive and growth, “-“ means negative and no growth.

### Denitrification capacity of strain *Alishewanella* sp. F2

**Figure 2:**
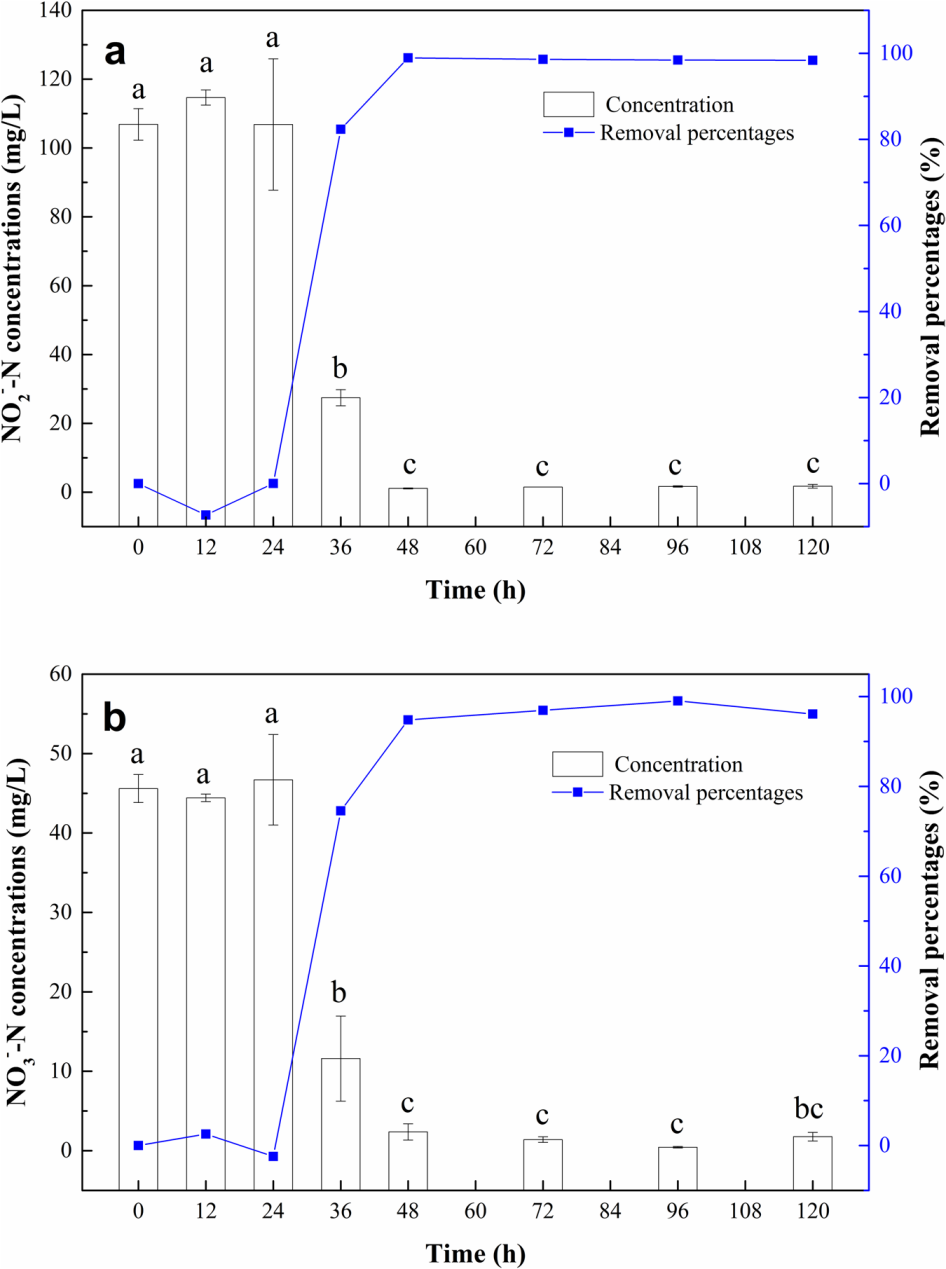
Removal of NO_2_^–^N (**a**) and NO_3_^–^N (**b**) using strain *Alishewanella* sp. F2 in denitrification medium. Values represent the mean of three replicates and error bars represent standard deviations. Columns containing different letters indicate significant differences among treatments at *p* = 0.05.

Strain *Alishewanella* sp. F2 was inoculated in a sterilized fresh denitrification medium containing NaNO_2_ as sole nitrogen source at a 5% inoculation size, and the concentrations of NO_2_^–^N and NO_3_^–^N in the culture solution were tested before and after the cultivation. The OD_600_ values of strain *Alishewanella* sp. F2 were measured, and the growth curve of strain *Alishewanella* sp. F2 was plotted (Fig. S4 in supplementary material). As shown in Fig. 2, the initial concentrations of NO_2_^–^N and NO_3_^–^N were 106.87 ± 4.58 mg/L and 45.60 ± 1.77 mg/L, respectively. There was basically no change in the concentrations of NO_2_^–^N and NO_3_^–^N within the first 24 h of cultivation, indicating that strain *Alishewanella* sp. F2 was in the growth adaptation period during the first 24 h. Strain *Alishewanella* sp. F2 exhibited inefficient denitrification capacity when it was in the stage of adapting to the new growth environment. After a 36 h cultivation, the concentrations of NO_2_^–^N and NO_3_^–^N in the medium decreased significantly (*p* < 0.05) to 27.45 ± 2.35 mg/L and 11.60 ± 5.35 mg/L with the removal percentages of 74.31% and 74.56%, respectively. Strain *Alishewanella* sp. F2 began to grow rapidly and entered the logarithmic growth phase (Fig. S4 in supplementary material) with obvious removal effects of NO_2_^–^N and NO_3_^–^N (Fig. 2). After a 48 h cultivation, the concentrations of NO_2_^–^N and NO_3_^–^N by strain *Alishewanella* sp. F2 decreased rapidly to 1.10 ± 0.14 mg/L and 2.37 ± 1.03 mg/L, with the corresponding removal percentages of 98.97% and 94.81%, respectively. During the 48−120 h cultivation, the removal percentages of NO_2_^–^N and NO_3_^–^N were above 98% and 94%, respectively. Strain *Alishewanella* sp. F2 entered a stable growth stage after 48 h and maintained high removal abilities of NO_2_^–^N and NO_3_^–^N until the end of 120 h. The efficient removal abilities of NO_2_^–^N and NO_3_^–^N of strain *Alishewanella* sp. F2 were achieved. During the cultivation process, the reduction of NO_2_^–^N and NO_3_^–^N concentrations was accompanied by gas generation, demonstrating that that strain *Alishewanella* sp. F2 can transform nitrites and nitrates into gas, thus achieving effective denitrification performance. For the control treatment without inoculating of strain *Alishewanella* sp. F2, the concentrations of NO_2_^–^N and NO_3_^–^N did not evidently change during the cultivation process (only 4% nitrite was transformed to nitrate).

### Salt tolerance of *Alishewanella* sp. F2

Strain *Alishewanella* sp. F2 was separated from seawater muddy water, which was a saline environment, therefore, it was suspected that strain *Alishewanella* sp. F2 was salt tolerant. To further verify the salt tolerance and its threshold of strain *Alishewanella* sp. F2, strain *Alishewanella* sp. F2 was cultivated in double-layer screening-isolation medium with different salinity gradients for 5 d. The growth of strain *Alishewanella* sp. F2 is shown in Table 3. After cultivation of 2 d, there was obvious colony growth on the double-layer screening-isolation medium with 0 g/L and 30 g/L NaCl concentrations, and there were trace colonies on the medium with 50, 70 and 100 g/L NaCl concentrations. After cultivation of 5 d, obvious colony growth was observed on the medium with 0, 30, 50, and 70 g/L NaCl concentrations, and trace colony growth was observed on the medium with 100 g/L NaCl concentration. The results indicate that strain *Alishewanella* sp. F2 can tolerate a NaCl concentration of up to 100 g/L, although extreme high salt stress (i.e., 100 g/L NaCl) lead to a slower growth of strain *Alishewanella* sp. F2 compared to lower salt stress (i.e., 0, 30, 50 and 70 g/L NaCl).

**Table 3:** Growth of strain *Alishewanella* sp. F2 in screening-isolation medium with different salinity gradients.

Cultivate time	NaCl concentration in screening-isolation medium (g/L)				
(d)	0	30	50	70	100
1	—	—	—	—	—
2	++	++	+	+	+
3	++	++	++	++	+
4	++	++	++	++	+
5	++	++	++	++	+

++: Obvious colonies appeared on the surface of the medium, indicating that strain *Alishewanella* sp. F2 grew normally;

+: A small number of colonies appeared on the surface of the medium, indicating that strain *Alishewanella* sp. F2 grew but with low growth rate;

-: No colonies appeared on the surface of the medium, indicating that strain *Alishewanella* sp. F2 did not grow.

### Denitrification capacity of strain *Alishewanella* sp. F2 in saline conditions

**Figure 3:**
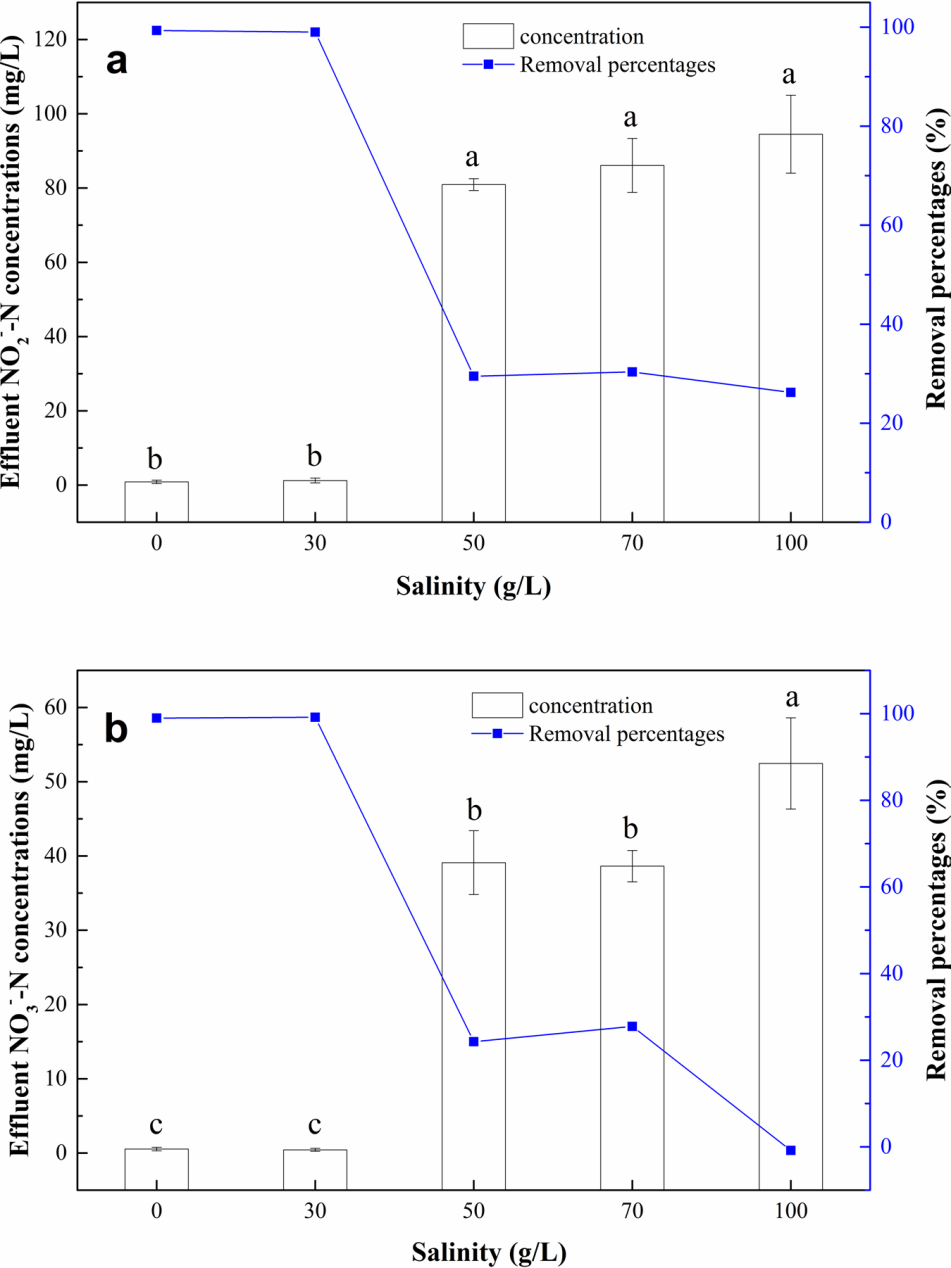
Effluent concentrations and removal percentages of NO_2_^–^N (**a**) and NO_3_^–^N (**b**) in denitrification medium with different salinity levels. Values represent the mean of three replicates and error bars represent standard deviations. Columns containing different letters indicate significant differences among treatments at *p* = 0.05.

The efficiencies of NO_2_^–^N and NO_3_^–^N removal by strain *Alishewanella* sp. F2 under different salinity treatment is shown in Fig. 3. The initial concentrations of NO_2_^–^N and NO_3_^–^N in denitrification medium of all the salinity treatments were maintained at 124.00 ± 8.72 mg/L and 52.36 ± 2.51 mg/L, respectively. Strain *Alishewanella* sp. F2 maintained high removal percentages for NO_2_^–^N and NO_3_^–^N when the NaCl concentration was at 30 g/L and below, with the NO_2_^–^N and NO_3_^–^N removal percentages of 99.00−99.34% and 98.99−99.17%, respectively. However, the removal ability of strain *Alishewanella* sp. F2 to both NO_2_^–^N and NO_3_^–^N was significantly (*p* < 0.05) inhibited by higher salinity treatments, i.e., NaCl concentration of 50−100 g/L in this study. In the medium with 50, 70, and 100 g/L NaCl concentrations, the removal percentages of NO_2_^–^N and NO_3_^–^N by strain *Alishewanella* sp. F2 were only 26.21–30.34% and 0–37.78%, respectively. The above observation indicates that the NaCl concentration higher than 50 g/L has a significantly (*p* < 0.05) negative effect on the denitrification capacity of strain *Alishewanella* sp. F2. Additionally, there was no significant difference in NO_2_^–^N removal percentage among salinity treatments of 50, 70, and 100 g/L NaCl. However, when the NaCl concentration was increased to 100 g/L, the removal percentage of NO_3_^–^N by strain *Alishewanella* sp. F2 was significantly reduced (*p* < 0.05) compared to 50 and 70 g/L NaCl treatments.

### Effect of pH, NaNO_2_ initial concentration and inoculation size on denitrification capacity of strain *Alishewanella* sp. F2

**Figure 4:**
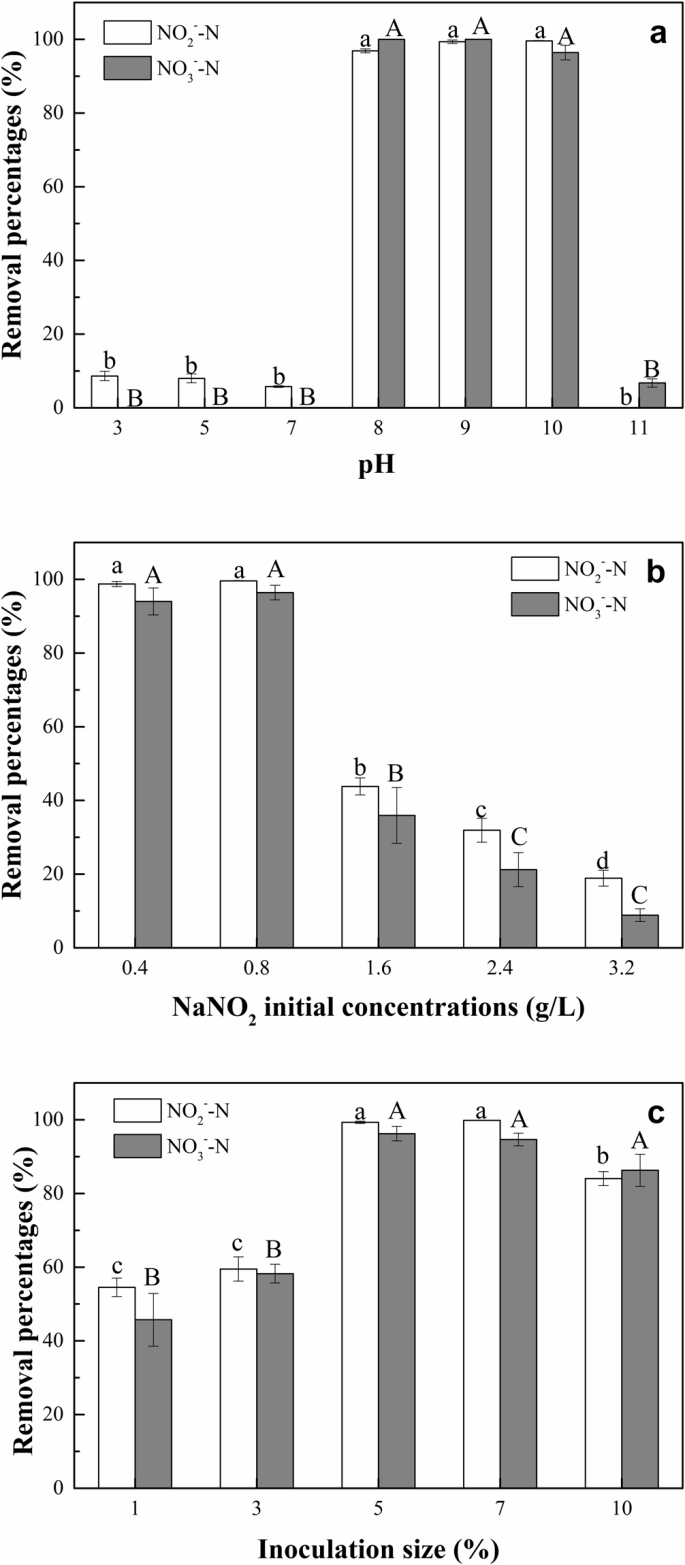
Denitrification capacity of strain *Alishewanella* sp. F2 as affected by different pH values (**a**), NaNO_2_ initial concentrations (**b**) and inoculation sizes (**c**) after a 5 d cultivation. Values represent the mean of three replicates and error bars represent standard deviations. Columns containing different letters indicate significant differences among treatments at *p* = 0.05.

The denitrification capacity of strain *Alishewanella* sp. F2 was affected by different pH value (Fig. 4a). When the pH was 3, 5 and 7, the removal percentages of NO_2_^–^N by strain *Alishewanella* sp. F2 was 5.76−8.65%, and there was no removal of NO_3_^–^N. When the pH was 8, 9 and 10, the removal percentages of NO_2_^–^N (96.85−100%) and NO_3_^–^N (96.41−100%) by strain *Alishewanella* sp. F2 were significantly increased (*p* < 0.05) compared to lower pH treatments (i.e., 3, 5 and 7). When the pH was 11, the NO_2_^–^N was not removed and only a small amount of NO_3_^–^N (6.74%) was removed.

The influence of different NaNO_2_ initial concentration on denitrification capacity of strain *Alishewanella* sp. F2 is shown in Fig. 4b. When the NaNO_2_ initial concentrations were 0.4 g/L and 0.8 g/L, the removal percentages of NO_2_^–^N and NO_3_^–^N were above 98% and 94%, respectively. With an increase in the NaNO_2_ initial concentration, the NO_2_^–^N and NO_3_^–^N removal abilities of strain *Alishewanella* sp. F2 were significantly decreased (*p* < 0.05). To be specific, the removal percentages of NO_2_^–^N and NO_3_^–^N by strain *Alishewanella* sp. F2 were significantly (*p* < 0.05) decreased when NaNO_2_ initial concentrations were in the range of 1.6−3.2 g/L, as compared with treatments of 0.4 g/L and 0.8 g/L. The average removal percentages of NO_2_^–^N and NO_3_^–^N with NaNO_2_ initial concentrations of 1.6−3.2 g/L were 18.90−43.81% and 8.85−35.94%, respectively.

As shown in Fig. 4c, the denitrification capacity of strain *Alishewanella* sp. F2 varied with different inoculation size. When the inoculation sizes were 1% and 3%, the NO_2_^–^N and NO_3_^–^N removal percentages were 54.53−59.50% and 45.73−58.24%, respectively. With an increase in the inoculation size, the NO_2_^–^N and NO_3_^–^N removal percentages of strain *Alishewanella* sp. F2 were significantly increased (*p* < 0.05). When the inoculation sizes were 5% and 7%, the average removal percentages of NO_2_^–^N and NO_3_^–^N were 99.33–99.84% and 94.66–96.23%, respectively. There was no significant difference in NO_2_^–^N removal when further increase the inoculation size to 10%. However, when the inoculation size was 10%, the NO_3_^–^N removal percentage by strain *Alishewanella* sp. F2 was significantly (*p* < 0.05) reduced compared to 5% and 7% treatments.

## Discussion

In recent years, many bacteria with denitrification capacity have been studied. Denitrifying bacteria do not have a specific taxonomy in microbial taxonomy and they are scattered among many genera of prokaryotes. In a highly saline environment, the growth and metabolism of most bacteria are apt to be inhibited and even lead to death^26^. General marine microorganisms grow in a saline environment of 10−30 g/L NaCl, and belong to slightly halophilic bacteria, while moderately halophilic bacteria can grow in a saline environment of 30−145 g/L NaCl^30^. A few halophilic denitrifying bacteria were found, mainly distributed in *Bacillus* and *Halomonas* bacteria, and *Halobacterium* and *Haloarcula* of archaea, etc.^31^. In particular, the halophilic bacteria that can survive in salt-free conditions are defined as salt-tolerant bacteria^32^. For example, a salt-tolerant bacterium strain *Bacillus hwajinpoensis* SLWX_2_ was screened from sea water, and the removal percentages of NO_2_^–^N and NO_3_^–^N at 24 h in a concentration of 30 g/L NaCl by a strain of *Bacillus hwajinpoensis* SLWX_2_ were 99.5% and 85.6%, respectively^33^. In this study, a salt-tolerant denitrifying bacterium strain *Alishewanella* sp. F2 was isolated from seawall muddy water and its salinity tolerance ranged from 0 to 70 g/L NaCl. It is reported for the first time that the *Alishewanella* sp. has the characteristic of salt tolerance. Strain *Alishewanella* sp. F2 is different from the previous reported salt-tolerant denitrifying bacteria, which indicates the diversity of denitrifying bacteria in nature, and is of great significance for enriching the knowledge of denitrifying bacterial ecology.

In anoxic conditions, nitrites and nitrates mainly undergo denitrifying processes, which are converted to gaseous products, e.g., N_2_, N_2_O or other biochemical reduction products^12,28^. Most available studies used the anaerobic incubator (e.g., Gas-pak Anaerobic Jar) to create an anaerobic environment^34,35^. However, an anoxic environment is necessary for the culturing of facultative anaerobes. Therefore, the submerged culture and sandwiching plate method, which can create an anoxic environment, was developed in this study, and the ideal target strain *Alishewanella* sp. F2 exhibiting excellent NO_2_^–^N and NO_3_^–^N removal ability in saline conditions was obtained.

Environmental conditions not only affect the growth and metabolism of bacteria, but also affect the denitrification capacity^36–38^. Optimal environmental conditions and nutrients are necessary for promoting microbial growth and metabolism and improving biological nitrogen removal^39^. A variety of factors (i.e., salinity, pH, initial nitrite concentration) was considered during the screening of denitrifying bacteria in this study. Exploring the optimal condition for growth and denitrification of strain *Alishewanella* sp. F2 will provide important guidance for practical application in environmental conditions.

The growth and reproduction of bacteria in a medium go through four phases, i.e. lag phase, exponential phase, stationary phase and death phase^40^. The denitrification process of strain *Alishewanella* sp. F2 mainly occurred between 24 to 48 h after cultivation, for both NO_3_^–^N and NO_2_^–^N (Fig. 2). Combining with the growth curve of strain *Alishewanella* sp. F2 (Fig. S4 in supplementary material), it can be concluded that the denitrification mainly occurred in the exponential phase (12−48 h), which is similar to the strains reported in previous studies^41,42^. There was no obvious change in the NO_2_^–^N and NO_3_^–^N concentrations during the lag phase (0−12 h), stationary phase (48 h later) and death phase (Fig. S4 in supplementary material and Fig. 2), indicating that no obvious denitrification process occurred during these periods. During the exponential phase, bacteria grow fastest and metabolize most vigorously and the energy and reducing power needed for cell synthesis are mainly consumed at this stage^43^. Therefore, the highest denitrification efficiency of strain *Alishewanella* sp. F2 was observed during the exponential phase.

*Alishewanella* is a genus of the class *Gammaproteobacteria* in the phylum *Proteobacteria*^44^. *Proteobacteria* is the largest phylum in the bacterial domain and is commonly found in a variety of environments. *Proteobacteria* is salt tolerant, and it is dominant under 0−18 g/L salinity, and the relative abundance increases with the increasing salinity levels^45^. In addition, *Gammaproteobacteria* can tolerate highly saline environments^45,46^. In this study, strain *Alishewanella* sp. F2 showed a high ability to remove NO_2_^–^N and NO_3_^–^N in medium with 0−30 g/L NaCl concentrations, reflecting the high salt tolerance of strain *Alishewanella* sp. F2 in the experimental system. Besides its high salt tolerance, strain *Alishewanella* sp. F2 can also adapt to a high alkali condition (pH of 8−10). In the condition of pH = 10 and 30 g/L NaCl concentration, the removal percentages of strain *Alishewanella* sp. F2 to NO_2_^–^N and NO_3_^–^N with initial concentrations of 123.17 ± 1.01 mg/L and 52.07 ± 1.16 mg/L were 99.00% and 99.17%, respectively (Fig. 3). Strain *Alishewanella* sp. F2 has the characteristics of strong adaptability, fast growth rate, high nitrogen removal efficiency, etc. Therefore, the discovery of strain *Alishewanella* sp. F2 and the evaluation of its nitrogen removal characteristics in highly saline environments can provide reference for the microbial nitrogen removal process. The denitrification capacity of strain *Alishewanella* sp. F2 under salt-alkali stress indicates that strain *Alishewanella* sp. F2 has a promising application prospect in the treatment of saline wastewater. The culture conditions, denitrification characteristics and large-scale applications in the application of strain *Alishewanella* sp. F2 are recommended for further study.

## Conclusion

Three denitrifying bacteria were isolated from seawall muddy water in Dalian city, Liaoning province, China. Strain F2 proved to be more effective in NO_2_^–^N and NO_3_^–^N removal than other strains, and was identified as *Alishewanella* sp., named *Alishewanella* sp. F2 (GenBank accession no. MN396708). Strain *Alishewanella* sp. F2 was deposited at the CGMCC on March 25, 2019, numbered CGMCC No: 17433.

Strain *Alishewanella* sp. F2 has a promising salt-tolerant denitrification capacity. The removal percentages of NO_2_^–^N and NO_3_^–^N by strain *Alishewanella* sp. F2 in a saline condition of 30 g/L NaCl were all above 99%. Besides, strain *Alishewanella* sp. F2 has an efficient denitrification capacity under high alkali conditions (pH of 8−10) and high initial nitrogen concentrations (NaNO_2_ of 0.4−0.8 g/L). In summary, strain *Alishewanella* sp. F2 is an efficient salt-tolerant denitrifying bacterium, which can be potentially applied in denitrification of saline wastewater in the future.

## Materials and Methods

### Sample collection and culture media description

Muddy water samples were collected from the estuary of an aquaculture wastewater stream in Dalian City, Liaoning Province, China (39°38′31′′ N, 122°58′19′′ E), and stored at 4 °C before further treatment in the Key Laboratory of Wetland Ecology and Environment, Chinese Academy of Sciences, China.

The culture media were described as follows: denitrification medium was composed of (g/L) CH_3_COONa 5, K_2_HPO_4_ 1, NaNO_2_ 0.8, CaCl_2_ 0.03, NaCO_3_ 1, FeSO_4_•7H_2_O 0.06, MgSO_4_•7H_2_O 0.2 and PH = 10. It was noteworthy that FeSO_4_•7H_2_O was added after the addition of deionized water to avoid oxidation of divalent iron (Fe^2+^) to ferric iron (Fe^3+^)^47^. Ingredients for the screening-isolation medium (pH = 10) and oblique tube preservation medium (pH = 10) were same as the denitrification medium but with 2% agar (m/v) added. All media were autoclaved at 121 °C for 30 min before applying.

### Enrichment, isolation and screening of bacterial strains

Two mL seawall muddy water samples were added to the 250 mL Erlenmeyer flasks containing 200 mL denitrification medium with three replicates, cultivated in a constant temperature incubator at 30 °C for 5 d. The flasks were sealed with parafilm to avoid gas exchange. Three successive transfers were carried out in fresh denitrification medium by subculturing 2 mL inoculum and incubating for 5 d for each time.

Following the last transfer of 5 d, the cultures were diluted 1000-fold. A 0.5 mL of each dilution was spread on screening-isolation medium in the glass Petri dish. Then an unsolified screening-isolation medium (<40 °C) was poured on the culture for air isolation. The Petri dishes were sealed, turned over and placed at 30 °C in a constant temperature incubator until clear colonies appeared. Isolated colonies were streaked onto new double-layer screening-isolation medium dishes, purified by repeated streaking.

### Denitrification capacity test of the isolated strains

The strains preserved on the preservation medium were inoculated into the denitrification medium and incubated for 5 d in hypoxic conditions at 30 °C. Each strain was inoculated into a fresh denitrification medium at a 5% inoculation size, and the flask mouth was sealed and statically cultivated at 30 °C for 5 d. During the incubation, the turbidity and gas production of the denitrification medium were observed and recorded. Finally, 10 mL liquid sample was taken periodically, 5 mL cultivated solution was taken at regular intervals, and centrifuged at 5000 r/min for 10 min, and the supernatant was then diluted and measured for NO_2_^–^N and NO_3_^–^N concentrations. The concentrations of NO_2_^–^N and NO_3_^–^N in water samples were determined by N-(1-naphthyl)-ethylenediamine spectrophotometry and naphthyl ethylenediamine hydrochloride spectrophotometry, respectively. The removal percentages of NO_2_^–^N and NO_3_^–^N were calculated to determine the denitrification capacity of strains.

In order to clarify the growth of bacteria and ensure the denitrification capacity, the test experiment was carried out when the bacteria were in logarithmic phase. A single bacterial colony was selected from the oblique tube preservation medium, inoculated using sterilizing inoculator, and cultivated in denitrification medium at 30 °C under anoxic condition. The turbidimetric method (OD_600_) was used to determine the growth of bacteria. The OD_600_ value of bacteria was determined every 12 h, and the growth curve of bacteria was drawn.

### Identification of strain F2

According to the results of denitrification capacity test (see Result), the denitrification capacity of strain F2 was more efficient than other strains. Therefore, we focused on only strain F2 in the following experiments including the salt tolerance test and the analysis of influencing factors. The morphology and the physiological and biochemical experiments of strain F2 were carried out according to the *Manual for Systematic Identification of Common Bacteria*^48^ and the *Bergey’s manual of systematic bacteriology*^49^. Using an Ezup column bacterial genomic DNA extraction kit (Sangon Co. Ltd., China), the total genome DNA of strain F2 was extracted by a conventional method. Using total DNA as a template, the genomic DNA of strain F2 was amplified by the polymerase chain reaction (PCR) thermal cycler (Mastercycler, Eppendorf Co. Ltd., German). The primers for PCR reaction were 16 S rDNA amplification universal primers. The forward primer was 27 F 5′-AGAGTTTGATCCTGGCTCAG-3′ and the reverse primer was 1492 R 5′-GGTTACCTTGTTACGACTT-3′. The composition of the PCR reaction system is as follows: 0.5 μL of template DNA, 1 μL of dNTP (mix), 2.5 μL of Taq Buffer (with MgCl_2_), 0.2 μL of Taq enzyme, 0.5 μL of primer F (10 μM), 0.5 μL of primer R (10 μM), and double distilled water to 25 μL. Under the following conditions, the amplification of PCR was completed: requires at 94 °C for 4 min, denaturation at 94 °C for 45 s, annealing at 55 °C for 45 s, extension at 72 °C for 1 min, 30 cycles, repair extension at 72 °C for 10 min and reaction was terminated at 4 °C. The PCR amplification product of 5 μL was detected by 1% agarose gel electrophoresis. The 16 S rDNA sequencing of recycled PCR products was carried out in Sangon Biotech (Shanghai, China) Co., Ltd. The sequence obtained by 16 S rDNA sequencing was compared and analyzed by using the BLAST at the National Center for Biotechnology Information (NCBI) (*http://:/*www.ncbi.nlm.nih.gov/). The phylogenetic tree of strain F2 was constructed by Neighbor-Joining method in MEGA 4.0 (Arizona State University, 2007) software, and the species of strain F2 were determined.

### Salt tolerance test of strain F2

The growth of strain F2 was observed while changing the salinity levels of screening-isolation medium. Five salinity levels of the screening-isolation medium were designed, i.e., NaCl concentrations of 0, 30, 50, 70, and 100 g/L, respectively. Strain F2 from the same concentration was streaked on each plate of the same area with designated salinity level and placed in an incubator at 30 °C for 5 d. Within each plate, the number of lines and the line spacing were ensured to be consistent. Each treatment was repeated three times to avoid the error caused by manual operation. The colony of strain F2 on the medium plate was determined for evaluating the tolerance of strain F2 to different salinity levels.

### Evaluation of the denitrification capacity of strain F2 under different salinity levels

The denitrification medium with different salinity levels was prepared by setting NaCl concentrations at 0, 30, 50, 70, and 100 g/L, respectively. Strain F2 was inoculated in the denitrification medium at a 5% inoculation size, and the flask mouth was sealed and statically cultivated at 30 °C for 5 d. The removal percentages of strain F2 to NO_2_^–^N and NO_3_^–^N under each respective salinity level were determined and calculated as described above.

### Effects of initial pH values, NaNO_2_ initial concentrations, and inoculation sizes on denitrification capacity of strain F2 in saline conditions

Strain F2 was cultivated in a fresh denitrification medium with 30 g/L NaCl concentration for 5 d until the culture solution was cloudy and there was gas produced. As influencing factors, initial pH (i.e., 3, 5, 7, 9, 10 and 11), NaNO_2_ initial concentrations (i.e., 0.4, 0.8, 1.6, 2.4 and 3.2 g/L) and inoculation sizes (v/v) (1, 3, 5, 7 and 10%) were observed for the effects on the NO_2_^–^N and NO_3_^–^N removal of strains. In this experiment, only each respective factor that tested was changed while the other conditions remained constant. After cultivation for 5 d, samples were taken to determine the concentrations of NO_2_^–^N and NO_3_^–^N.

### Statistical analysis

All results were presented as the average of three independent experiments. The data presented in the figures were expressed as means ± standard deviation. Means between different treatments were compared by one-way ANOVA with *Tukey’s* test at the significance level of 0.05. All statistical analyses were performed by using Microsoft Office Excel 2007 and SPSS 22.0 for Windows system. All graph design was carried out by Origin 9.1 for Windows system.

## Supplementary information

Supplemental Information.

## Data Availability

The datasets generated during and/or analysed during the current study are available from the corresponding author on reasonable request.
