# Supplementary material for: Isolation and characterization of a salt-tolerant denitrifying bacterium Alishewanella sp. F2 from seawall muddy water
Source: Sci Rep. 2020 Jun 19;10:10002. doi: 10.1038/s41598-020-66989-5 (PMC7305158; doi:10.1038/s41598-020-66989-5)
Supplement: Supplementary file 1 — Supplemental Information. [file 41598_2020_66989_MOESM1_ESM.doc]

### Supplemental Information for

**Isolation and characterization of a salt-tolerant denitrifying** **bacterium *Alishewanella* sp. F2 from seawall muddy water**

Rui Cheng^1,2,3^, Xinyi Wang^1,3,4^, Hui Zhu^1,3,*^, Baixing Yan^1,3^, Brian Shutes^5^, Yingying Xu^6^, Baorong Fu^4^, Huiyang Wen^1,3^

^1^ Key Laboratory of Wetland Ecology and Environment, Northeast Institute of Geography and Agroecology, Chinese Academy of Sciences, Changchun 130102, P R China.

^2^ University of Chinese Academy of Sciences, Beijing 100049, P R China.

^3^ Jilin Provincial Engineering Center of CWs Design in Cold Region & Beautiful Country Construction, Changchun 130102, P R China.

^4^ School of Environment, Liaoning University, Shenyang 110036, P R China.

^5^ Urban Pollution Research Centre, Middlesex University, Hendon, London, NW4 4BT, UK.

^6^ Key Laboratory of Songliao Aquatic Environment, Ministry of Education, Jilin Jianzhu University, Changchun 130118, P R China.

^*^ Corresponding author. Email: zhuhui@iga.ac.cn

**
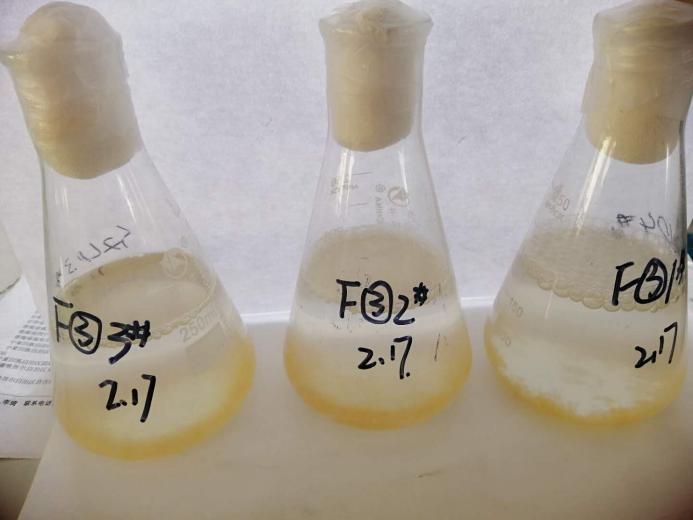
**

**Fig. S1** Enrichment culture of each strain (strain F3, F2 and F1 from left to right).


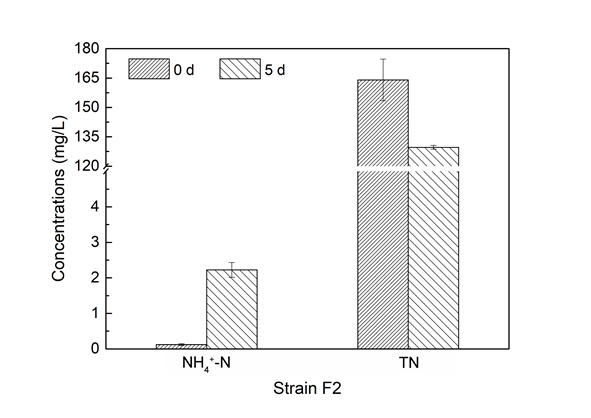


**Fig. S2** Changes of NH_4_^+^-N and TN in the culture of strain F2.


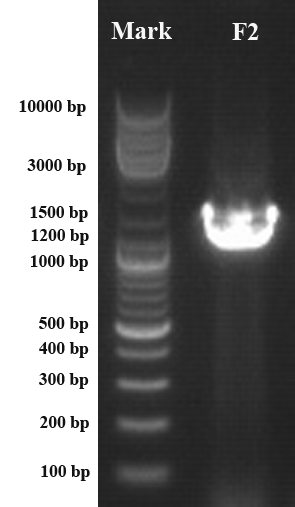


**Fig. S3** Amplification of 16S rDNA gene of strain F2 by PCR.

**

**

**Fig. S4** Growth of strain *Alishewanella* sp. F2 in the denitrification medium. Values represent the mean of three replicates and error bars represent standard deviations.
